# Wearable sensors to predict improvement following an exercise intervention in patients with knee osteoarthritis

**DOI:** 10.1186/s12984-017-0309-z

**Published:** 2017-09-12

**Authors:** Dylan Kobsar, Sean T. Osis, Jeffrey E. Boyd, Blayne A. Hettinga, Reed Ferber

**Affiliations:** 1 Faculty of Kinesiology, University of Calgary, 2500 University Dr NW, Calgary, AB T2N 1N4 Canada; 2 Department of Computer Science, University of Calgary, Calgary, AB Canada; 3 Faculty of Nursing, University of Calgary, Calgary, AB Canada; 4 Running Injury Clinic, Calgary, AB Canada

**Keywords:** Accelerometers, Wearable sensors, Gait analysis, Biomechanics, Knee osteoarthritis, Rehabilitation, Machine learning

## Abstract

**Background:**

Muscle strengthening exercises consistently demonstrate improvements in the pain and function of adults with knee osteoarthritis, but individual response rates can vary greatly. Identifying individuals who are more likely to respond is important in developing more efficient rehabilitation programs for knee osteoarthritis. Therefore, the purpose of this study was to determine if pre-intervention multi-sensor accelerometer data (e.g., back, thigh, shank, foot accelerometers) and patient reported outcome measures (e.g., pain, symptoms, function, quality of life) can retrospectively predict post-intervention response to a 6-week hip strengthening exercise intervention in a knee OA cohort.

**Methods:**

Thirty-nine adults with knee osteoarthritis completed a 6-week hip strengthening exercise intervention and were sub-grouped as Non-Responders, Low-Responders, or High-Responders following the intervention based on their change in patient reported outcome measures. Pre-intervention multi-sensor accelerometer data recorded at the back, thigh, shank, and foot and Knee Injury and Osteoarthritis Outcome Score subscale data were used as potential predictors of response in a discriminant analysis of principal components.

**Results:**

The thigh was the single best placement for classifying responder sub-groups (74.4%). Overall, the best combination of sensors was the back, thigh, and shank (81.7%), but a simplified two sensor solution using the back and thigh was not significantly different (80.0%; *p* = 0.27).

**Conclusions:**

While three sensors were best able to identify responders, a simplified two sensor array at the back and thigh may be the most ideal configuration to provide clinicians with an efficient and relatively unobtrusive way to use to optimize treatment.

**Electronic supplementary material:**

The online version of this article (10.1186/s12984-017-0309-z) contains supplementary material, which is available to authorized users.

## Background

Osteoarthritis (OA) is the most common musculoskeletal disease and will be diagnosed in nearly half of all people at some point in their life [1]. Specifically, knee OA accounts for more mobility disabilities in people over the age of 65 than any other medical condition [2]. While there is no known cure, muscle strengthening exercises are considered a mainstay in the management of knee OA [3]. Many patients report improvements in pain and function with muscle strengthening interventions, but the extent to which these improvements occur can greatly vary between individuals [4]. This heterogeneity in treatment response has led to a recent shift towards personalized medicine and the identification of those patients who respond to treatment (responders).

There is growing evidence supporting the need to predict outcomes to exercise interventions in knee OA and identify responders using baseline data. Previous research has demonstrated various clinical measures, including pain and function, can be predictive of response to exercise in OA [5, 6]. However, the association between subjective clinical measures and an individual’s response to treatment appears to be limited and inconsistent [7]. Preliminary findings have also suggested that biomechanical factors, such as varus thrust [8] and knee stability [9], may be predictive of response to exercise interventions in OA. Moreover, the integration of both objective biomechanical data and subjective clinical measures may further improve clinical prediction models. For example, previous research by our group [10] demonstrated that a unique combination of subjective patient reported outcome (PRO) measures and objective gait kinematic data collected from a three-dimensional (3D) motion capture system could successfully predict treatment outcome for knee OA patients with a classification accuracy of 85%. Specifically, individuals with knee OA whose baseline, pre-treatment data included low self-reported function, in combination with atypical hip frontal plane kinematics, responded best to the hip strengthening 6-week intervention. These findings demonstrated that baseline PRO measures and objective biomechanical gait data can effectively predict an individual’s treatment response to an exercise intervention. However, many clinicians do not have access to 3D motion capture technology necessary to collect biomechanical gait data. Wearable sensors may effectively bridge this gap by providing a clinically accessible alternative to acquire relevant gait data.

Wearable inertial sensors, such as accelerometers, are increasingly popular alternatives to 3D motion capture systems given their size, cost, and ease of use. However, their clinical impact up to this point has remained limited [11]. While research has demonstrated the validity [12] and reliability [13] of these devices, there continues to be a disconnect between the use of wearable sensors in a research setting and their potential clinical impact [11, 14]. Therefore, we postulate that a limited number of small and affordable wearable sensors can replicate previous research findings from Kobsar et al. [10], and thereby serve as an accessible source for the objective data necessary to provide an evidence-based approach regarding the prediction of treatment response for patients with knee OA.

The primary objective of this study was to determine if pre-intervention multi-sensor accelerometer data and PRO measures can retrospectively predict post-intervention response to a 6-week hip strengthening exercise intervention in a knee OA cohort. Specifically, we asked the following research questions; (i) what is the best placement of a single accelerometer for predicting post-intervention response, and (ii) what is the best combination of multiple accelerometers to predict post-intervention response? It was hypothesized that (i) the thigh accelerometer would achieve the best predictive accuracy of any single sensor, however (ii) the combination of all four sensors together would lead to a significantly better classification result.

## Methods

### Subjects

**Fig. 1:**
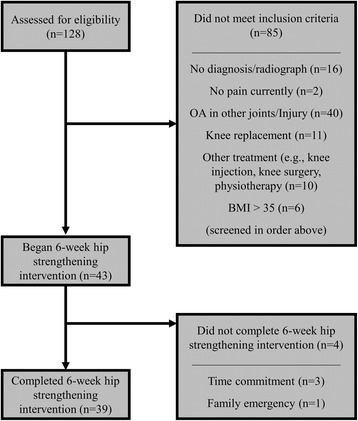
Diagram of patient screening and flow throughout study

A new cohort of 43 adults ≥40 years of age, similar to our previous work [10], were recruited for this study. All participants were radiographically diagnosed with knee OA, able to walk without assistive devices, and had no physical or medical condition for which the testing protocol would be contraindicated. Knee OA patients were excluded if they; [1] were diagnosed with severe knee OA and had been recommended by a physician for total knee arthroplasty, [2] were currently undertaking physiotherapy or other conservative management practices, including corticosteroid injections, [3] had taken oral corticosteroids or anti-inflammatories in the 24 h prior to testing, [4] had systemic arthritic conditions, or [5] had a body mass index (BMI) > 35 kg/m^2^. Screening and patient flow diagram is shown in Fig. 1. This research was approved by the Conjoint Health Research Ethics Board at the University of Calgary and all participants provided written, informed consent prior to participating.

### Procedure and device

**Fig. 2:**
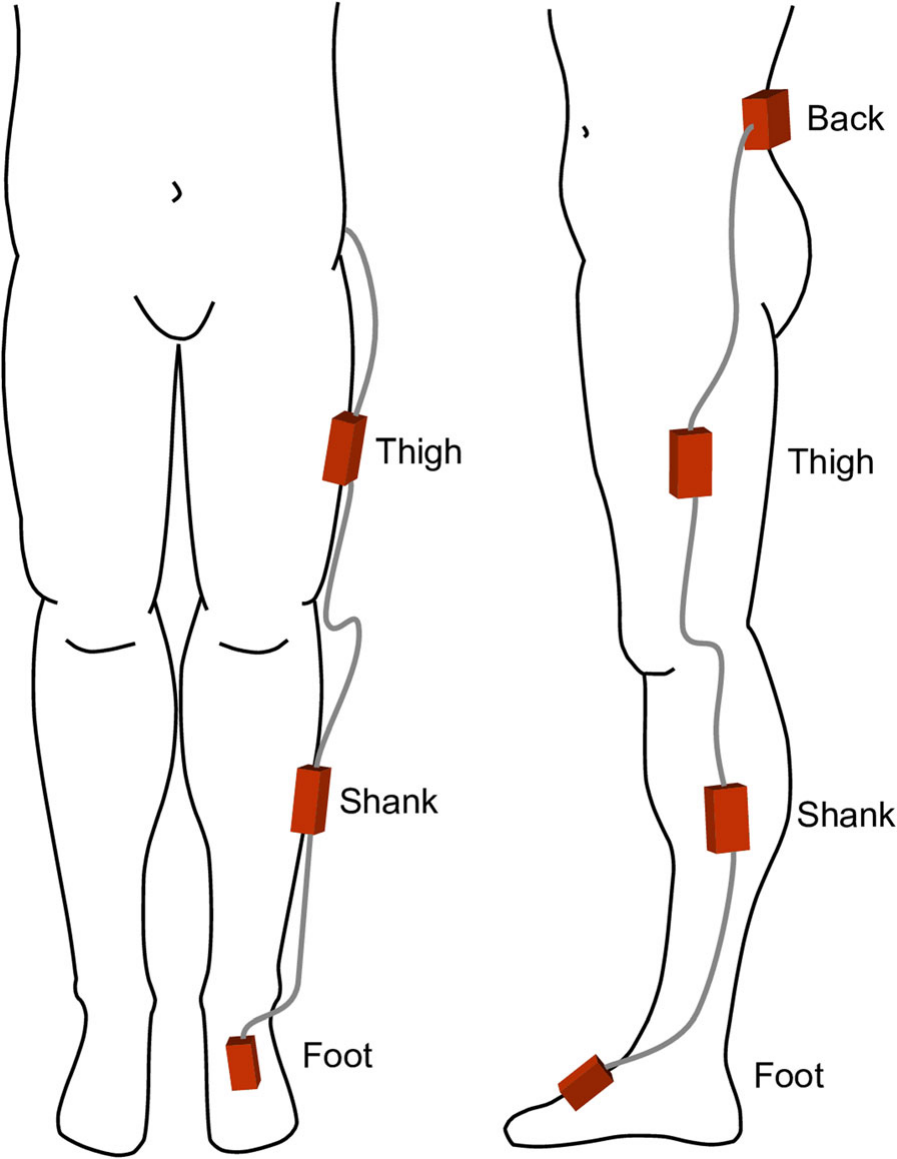
Positioning of inertial sensors on the back, thigh, shank, and foot of the most affected side of knee osteoarthritis patients

Prior to beginning the exercise intervention, a gait analysis was completed on each participant. The participants walked on a level treadmill (Bertec, Columbus OH, USA) for 3–5 min to acclimatize to the set-up and identify their preferred pace. Following this period, 60s of data were collected for analysis. Gait data were recorded using four synchronized inertial measurement units (iNEMO inertial module, STmicroelectronics, Geneva, Switzerland) custom developed by Alberta Centre for Advanced MNT Products (Calgary, Canada). See Fig. 2. A sensor (5.5 cm × 3.5 cm × 1.5 cm) was placed on the dorsum of the foot near the base of the third metatarsal and held firmly in place with double-sided tape under the sensor and single-sided athletic tape over sensor. A second sensor (8 cm × 3.5 cm × 1.5 cm) was placed on the shank at the midpoint between the lateral knee joint line and the lateral malleolus. A third sensor (8 cm × 3.5 cm × 1.5 cm) was placed on the thigh at the midpoint between the greater trochanter and the lateral knee joint line. The shank and thigh sensors were carefully placed at the midpoint between these landmarks by measuring with a vinyl tape measure to ensure consistently in placement. All three lower limb sensors were placed on the most affected leg of the participant. Finally, a larger, master sensor (10.5 cm × 6 cm × 3 cm) was placed on the lower back directly between and superior to the posterior superior iliac spines, at the approximate height of L3. This master sensor not only collected gait data, but stored all synchronized data from the four sensors. Each sensor collected 3D accelerometer (±16 g linear accelerations) and gyroscope (±2000°/s angular velocities) data sampling at 100 Hz. This protocol for placing sensors has been shown to achieve highly reliable between day measurements in patients with knee OA (average coefficient of multiple determination of 0.96 ± 0.05) [13].

In addition to the gait analysis, participants also completed a Knee injury and Osteoarthritis Outcome Score (KOOS). The KOOS is a 42-item self-administered knee-specific standardized questionnaire (5 Likert boxes) designed to address five subscales; pain, symptoms, function in daily living (ADL), sport and recreation function, and knee related quality of life (QoL) with a high level of reliability [15]. The KOOS is reported as a normalized score calculated for each subscale (100 indicating no symptoms and 0 indicating extreme symptoms) [16]. In the current study, only four subscales were examined (pain, symptoms, ADL, and QoL) as many of the sport and recreation function questions did not apply to the patients. Participants completed the KOOS prior-to and following the exercise intervention.

### Intervention

Following baseline testing, participants completed a 6-week therapist-directed hip muscle strengthening program. The exercise protocol focused on the hip abductors, hip flexors, hip external rotators, and core stability using Thera-Band (The Hygenic Corporation, Akron OH, USA) elastic bands. The exercises were shown to each participant by a Board Certified Athletic Therapist who oversaw one exercise session per week to monitor pain, compliance, and technique. The exercises given at each weekly training session were standardized based on the strengthening program (see Additional file 1), but the individual progression of the exercises (e.g., sets, repetitions, band resistance) were at the discretion of the Athletic Therapist based on patient feedback and symptoms during rehabilitation. Participants were asked to perform the exercises daily and record the days they completed the exercises. The average (standard deviation) number of days per week in which the exercises were completed by the participants was 5.3 (0.9). The strengthening program was based on a protocol (see Additional file 1) that has been shown to be effective for improving symptoms in patients suffering from patellofemoral pain syndrome [17] and knee OA [10].

### Responders

**Fig. 3:**
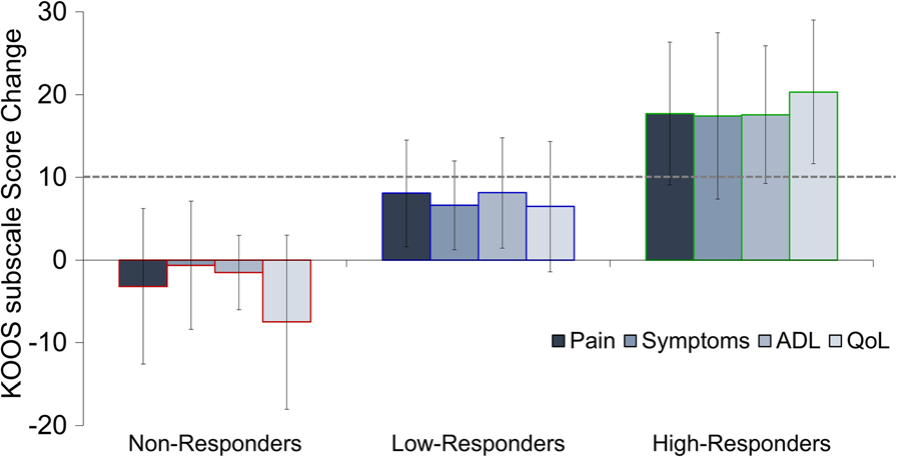
Absolute change in Knee Injury and Osteoarthritis Outcome Score (KOOS) subscale scores following 6-week intervention, organized by responder sub-groups. The dotted line represents the minimal clinically important improvement [18]. Abbreviations: ADL = Function in Daily Living; QoL = Knee Related Quality of Life.. Note: KOOS is measured on a scale of 100, with 100 best (e.g., no pain) and 0 being worst (e.g., most pain imaginable)

Participants were sub-grouped based on their change in PRO data (KOOS subscales; pain, symptoms, ADL, and QoL) following the 6-week exercise intervention. The effect size (Cohen’s d) of the intervention for each participant was averaged across all four KOOS subscales to label participants as Non-Responders (*n* = 10; effect size < 0.2), Low-Responders (*n* = 20; 0.2 ≤ effect size < 0.8), or High-Responders (*n* = 9; effect size ≥0.8) [10]. Four participants did not complete the intervention, with three reporting issues with time commitment, and one reporting a family emergency (Fig. 1). Demographics for the three sub-groups, as well as the four dropout participants, are reported in Table 1. The absolute change in KOOS subscales following the 6-week exercise intervention for each responder sub-group, along with the minimal clinically important improvement [18] are shown in Fig. 3.

**Table 1:** Demographics of knee osteoarthritis patients sub-grouped by response to the exercise intervention

	Groups	n	Mean (SD)	95% CI
Gait Speed (m/s)	Non-Responders	10	1.06 (0.16)	0.94–1.18
	Low-Responders	20	1.11 (0.09)	1.06–1.15
	High-Responders	9	1.19 (0.10)	1.11–1.26
	Total	39	1.11 (0.12)	1.07–1.15
	Dropouts	4	1.14 (0.07)	1.02–1.25
Age (years)	Non-Responders	10	60 (8)	54–65
	Low-Responders	20	57 (7)	53–60
	High-Responders	9	59 (10)	52–67
	Total	39	59 (8)	55–61
	Dropouts	4	57 (3)	47–67
BMI (kg/m^2^)	Non-Responders	10	26.0 (4.5)	22.8–29.3
	Low-Responders	20	26.4 (3.6)	24.8–28.1
	High-Responders	9	27.6 (3.7)	24.8–30.4
	Total	39	26.6 (3.8)	25.4–27.8
	Dropouts	4	24.1 (3.6)	18.3–30.0

No significant differences were observed between sub-groups

### Data processing

**Fig. 4:**
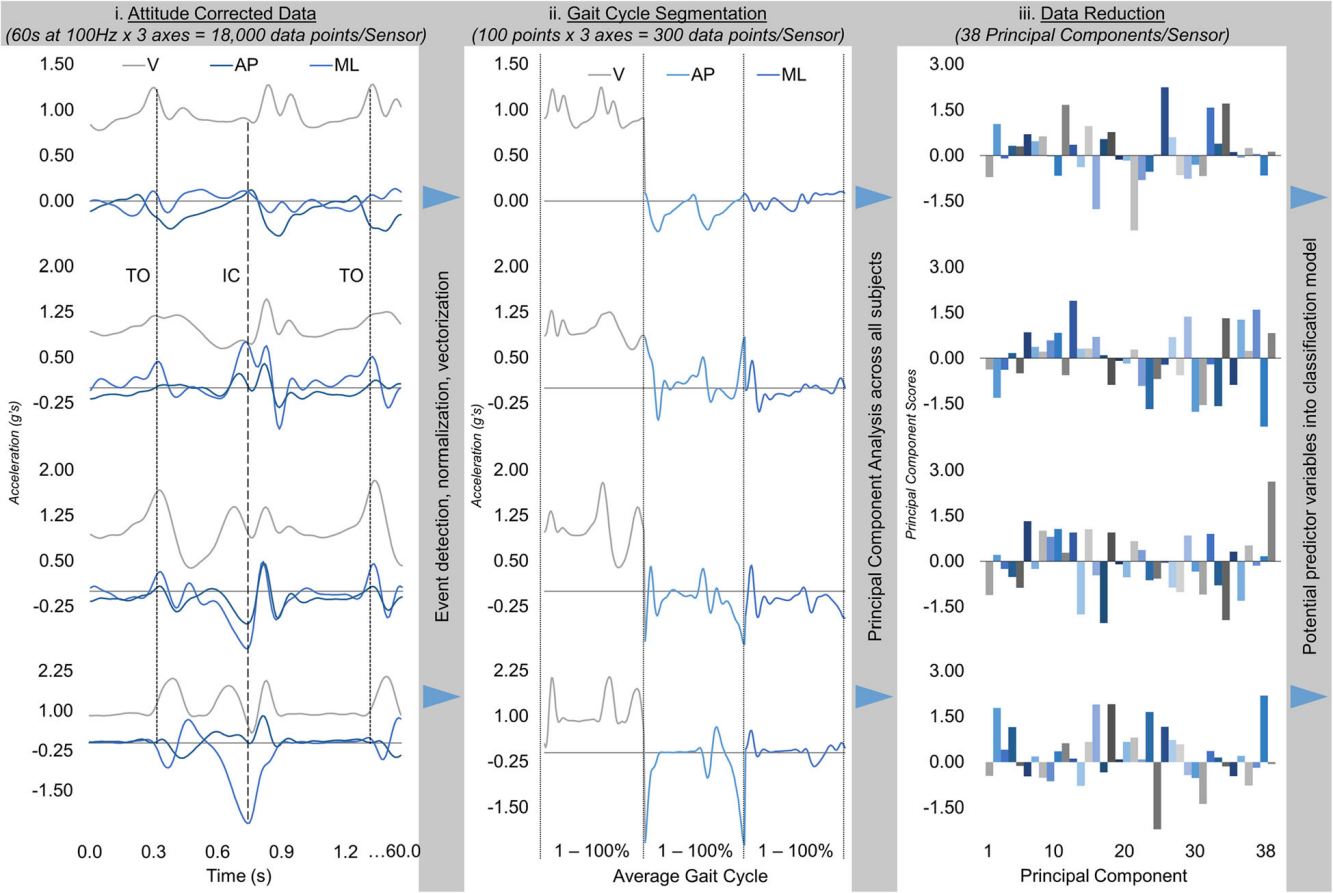
Representative example of accelerometer data processing steps (**i**) attitude correction, (**ii**) gait cycle segmentation, and (**iii**) data reduction. The four sensor placement signals are ordered from back (top) to foot (bottom)

All data processing was performed using MATLAB 9.0 (The MathWorks Inc., Natick, MA) with statistical analyses performed in IBM SPSS Statistics 24 (SPSS Inc., Armonk NY, USA). Data from each sensor were initially filtered with a 10 Hz low-pass 4th order recursive Butterworth filter. Linear acceleration data then underwent three steps of data processing before being used for classification of knee OA responder sub-groups. These three data processing steps were; (i) attitude correction, (ii) gait cycle segmentation, and (iii) data reduction (Fig. 4).

First, each sensor underwent a (i) static attitude correction procedure to align each accelerometer with the global vertical and horizontal planes. While extreme care was taken in placing the sensors consistently and accurately on the back, thigh, shank, and foot, applying an attitude correction has been shown to improve the reliability of the 3D linear accelerations [13]. This alignment was achieved by computing the initial orientation of each sensor from the static gravitational vector measured during a 5 s static standing trial prior to the walking trial. A rotation matrix was then derived from this initial orientation and applied to all subsequent linear accelerations obtained during the 60 s of walking trial data [13, 19], thereby mathematically aligning the sensors with global frame during the walking trial. A representative example of attitude corrected accelerometer data can be seen in Fig. 4i.

Following the attitude correction, the linear acceleration data were (ii) segmented into gait cycles and time-normalized to 60 points for stance phase and 40 points for swing phase for each axis in all sensors. To accurately segment into gait cycles, initial contact and toe-off gait events were obtained from the angular velocity about the mediolateral axis recorded by the foot sensor. These events were detected using custom MATLAB 9.0 (The MathWorks Inc., Natick, MA) algorithms, similar to previous work [20, 21]. Representative examples of these events are shown on the attitude corrected acceleration waveforms in Fig. 4i. Initial contact and toe-off gait events were then used to segment the linear acceleration data at each sensor into time-normalized (60% stance; 40% swing) gait cycles. The average vertical, anteroposterior, and mediolateral linear acceleration waveform was computed from the gait cycles and concatenated into a 300-point vector, creating a 39 × 300 matrix (subject x average 3D linear accelerations) for each sensor (Fig. 4ii).

Finally, (iii) a principal component analysis (PCA) was used as a data reduction technique [22, 23]. A PCA approach has been used as a common data reduction technique that summarizes the variability in a set of potentially correlated data by creating a new set of linearly uncorrelated variables (i.e., principal components) to maximize the variability in the original data [23, 24]. Following normalization of each sensor’s 39 × 300 gait data matrix, a PCA transformed each sensor’s data into a reduced set of 38 variables (i.e., n-1 principal components) in a 39 × 38 matrix (subject x principal component scores) which retained 100% of the variation in the original dataset. These reduced pre-intervention gait data were then used as potential features in classifying the knee OA responder sub-groups (Fig. 4iii).

### Classification of responder sub-groups

Responder sub-groups were classified using a linear discriminant analysis and a sequential forward feature selection technique. A linear discriminant analysis is used to linearly combine multiple independent variables into k-1 composite variables, called discriminant functions (where k equals the number of responder groups), that best separate groups or discriminate group membership [22]. The ability of the discriminant functions (derived from pre-intervention data) to separate responder sub-groups (post-intervention response) was assessed using a 10-fold cross-validation error [25] across all possible combinations of sensor arrays. Given that four sensors were used in the current study, there are 15 unique combinations of sensor data (i.e., feature sets). These 15 feature sets consisted of four single-sensors sets used to determine the single best sensor for classification, as well as all 11 possible combinations of 2–4 sensors to determine the optimal array of sensors. For each feature set, pre-intervention gait data (e.g., principal component scores) from the sensor(s) were combined with pre-intervention KOOS data (pain, symptoms, ADL, and QoL subscale scores) to create the full set of potential features, which were then reduced to a limited subset via the sequential forward feature selection technique. This feature selection technique began with no predictor variables and sequentially added a single variable that produced the greatest error reduction in the model until adding any additional variable did not reduce this error [26]. Ten iterations of this process were conducted, resulting in a 10 × 10-fold cross-validation process [25], which allowed for statistical comparison of the classification accuracies aligned with our research questions for; (i) a single accelerometer and (ii) the best 2, 3, or 4 accelerometers arrays, using an analysis of variance with Fisher’s Least Significant Difference test (α = 0.05).

## Results

**Fig. 5:**
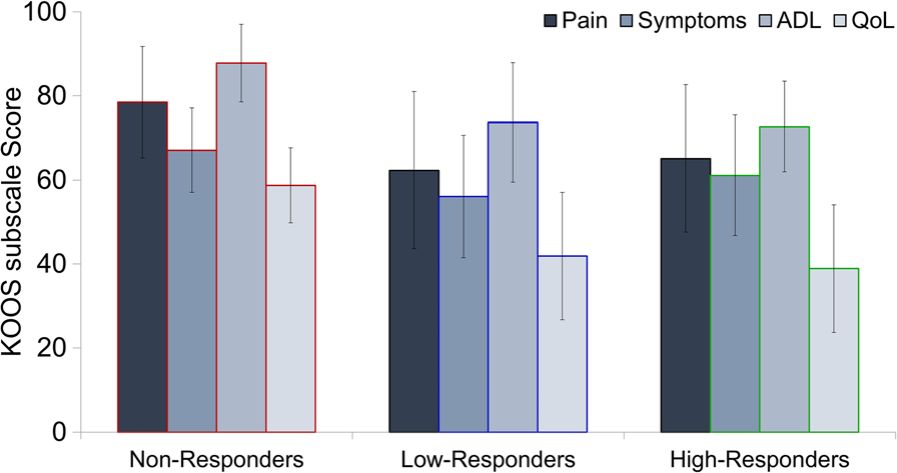
Baseline Knee Injury and Osteoarthritis Outcome Score (KOOS) subscale scores. Abbreviations: ADL = Function in Daily Living; QoL = Knee Related Quality of Life.. Note: KOOS is measured on a scale of 100, with 100 best (e.g., no pain) and 0 being worst (e.g., most pain imaginable)

**Fig. 6:**
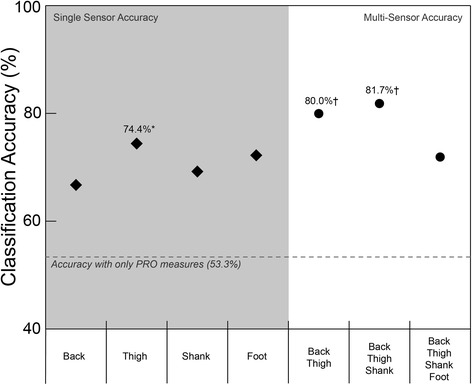
Classification accuracy for single accelerometer placements (diamond) and the best 2, 3, and 4 sensor arrays (circles). *Significantly greater than all other single sensor arrays. †Significantly greater than all other sensor arrays

**Fig. 7:**
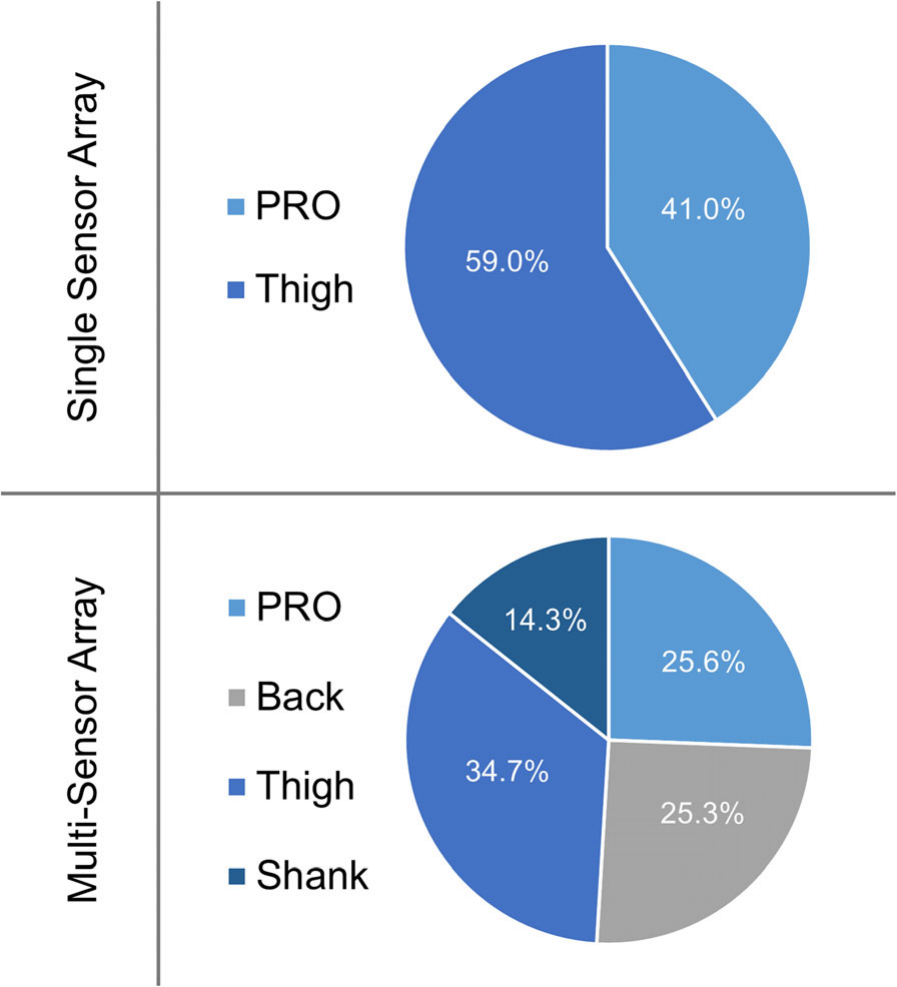
Relative importance (i.e., proportion of loading in model out of 100%) of the gait data and patient reported outcome (PRO) measures in the best single sensor (top) and multi-sensor (bottom) classification models, based on sum of squared coefficient loadings in discriminant analysis

**Fig. 8:**
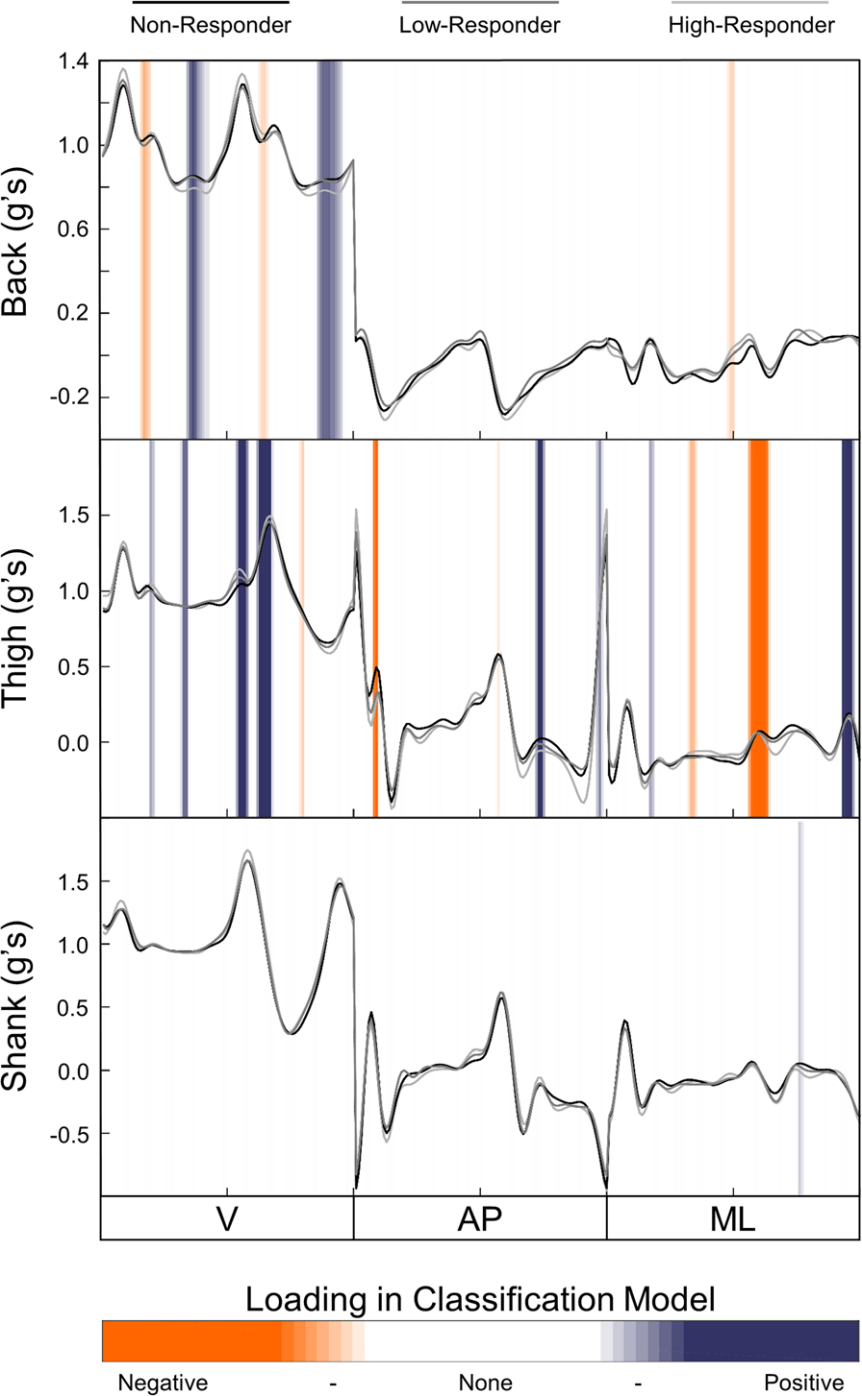
Mean acceleration waveforms of responder sub-groups, with relative positive (blue) and negative (orange) loading of gait principal components from the back, thigh, and shank sensors used in the three sensor classification model

Responder sub-groups were not significantly different in gait speed (*p* = 0.07), age (*p* = 0.55), or BMI (*p* = 0.66). Baseline PRO measures across responder sub-groups are shown in Fig. 5. When baseline gait data is combined with PRO data, the single best placement of the accelerometer was the thigh (Fig. 6), which had an average (standard deviation) 10-fold cross-validation classification accuracy of 74.4 (0.02)%, and was significantly better than the back (66.7 (0.05)%; *p* < 0.01), shank (69.2 (0.02)%; p < 0.01), and foot (71.4 (0.02)%; *p* = 0.03). This classification model included 9 total baseline variables (see Table 2); 6 thigh sensor gait PCs and 3 PRO measures (KOOS subscales; pain, symptoms, QoL), with the gait variables being the most important in the model (Fig. 7). The best combination of multiple sensors was the back, thigh, and shank, which displayed an 81.7 (0.04)% classification accuracy. This three sensor array was significantly different from all other single and multi-sensor configurations (p < 0.01), except for the combination of the back and thigh sensors (80.0 (0.02)%; *p* = 0.27; Fig. 5). The classification model using the back, thigh, and shank sensors included 9 total variables; 3 back sensor gait PCs, 2 thigh sensor gait PCs, 2 shank sensor gait PCs, 2 PRO measures (KOOS subscale pain and QoL), with the gait variables at the thigh being most important in the model (Fig. 7). The relative loading of the gait PCs at the back, thigh, and shank in the three sensor classification model can be visualized in Fig. 8.

**Table 2:** Variables in the best one, two, and three sensor classification models

	Thigh	Back, Thigh	Back, Thigh, Shank
Gait PCs	T1, T7, T14, T25, T38	B4, B16, T25, T38	B2, B4, B36, T7, T38, S8, S31
KOOS Subscales	Pain, Symptoms, QoL	Pain, QoL	Pain, QoL
Classification Accuracy	74.4%	80.0%	81.7%

*Abbreviations*: *Gait PC* Gait principal component numbers from *B* back, *T* thigh, *S* shank, *QoL* knee related quality of life

## Discussion

The purpose of this study was to retrospectively use pre-intervention accelerometer data and PRO measures to predict post-intervention response to exercise in knee OA patients. To our knowledge, this is the first study to use wearable sensors and PRO measures to predict treatment success in knee OA. In support of our first hypothesis, the thigh sensor proved to be the best location for a single sensor to predict response to exercise. On the other hand, it was not the combination of all sensors that achieved the best classification accuracy as we hypothesized, but the combination of only the back, thigh, and shank sensors. Moreover, this three-sensor array was not significantly different from the combination of just the back and thigh sensors, suggesting a two-sensor solution is all that is necessary for providing clinicians with an accessible system to make evidence-based decisions regarding optimal treatment for patients with knee OA.

Our main finding suggests that the thigh is the most effective placement for a single sensor when determining response to a hip strengthening exercise intervention in knee OA patients. The use of linear accelerations measured at the thigh in combination with PRO measures provided our model with a classification accuracy of 74.4%. This cross-validated accuracy was nearly 25% greater than the maximum chance criterion of 51%, based on the distribution of responder sub-groups. These findings support our hypothesis and previous findings using 3D motion capture technology [10]. Similar to the current results, we found hip joint kinematic patterns to be most important in classifying a comparable cohort of knee OA patients as responders to a similar hip strengthening exercise intervention [10]. These findings add to a growing body of evidence suggesting that the movement and control of the thigh and/or knee during walking can provide objective information that is useful in identifying responders and non-responders to a hip strengthening exercise intervention [8-10]. Nevertheless, including additional gait data may significantly improve our ability to classify responders.

Gait data from three sensors located at the back, thigh, and shank achieved the greatest overall classification accuracy. The linear accelerations measured at these three locations, combined with PRO measures (e.g., pain and QoL), provided our model with a classification accuracy of 81.7%. This classification accuracy demonstrated by the three sensors was similar to the 85% accuracy we demonstrated in research using 3D motion capture technology [10]. In both instances, while gait data obtained proximal to the knee was most important, data obtained distal to the knee helped to further improve discriminatory power. Much of the recent research on OA has suggested that there is not a single systematic change in gait (e.g., increased knee adduction moment), but rather a range of potential compensations that can occur across the hip, knee, and ankle [27, 28]. The utility of data collected at multiple segments/joints for identifying responder sub-groups in our current and previous [10] work supports this concept.

While examining complex gait patterns across multiple lower limbs joints can develop more detailed and effective classification models, it can also make it more difficult to find a simple, clinically interpretable relationship among the high-dimensional data in the models. The information presented in Figs. 5, 6 and 7 was meant to provide insights as to what type of data may be important to our classification problem. Overall, these figures reveal that while both gait and PRO data are important, it is the gait data, specifically thigh linear accelerations, that was most important in all classification models. This interpretation is a simplified, but clinically relevant finding that can directly help clinicians make evidence-informed decisions regarding treatment response for knee OA patients. However, to further identify more complex and detailed features in these gait patterns requires an in-depth analysis of the accelerometer signals, grounded in the loading of the features (PCs) used in the classification models. For example, Fig. 8 presents the average accelerometer gait patterns of each responder sub-group, while also illustrating the relative loadings of the PC features used in the three-sensor classification model in order to highlight the areas of the gait waveforms that are most important to the classification problem. As previously discussed, this high-dimensional data becomes more difficult to interpret, but visualizing the loading of gait features in the classification model, either negative in orange or positive in blue, can provide further insight into this problem. Specifically, we can see the highest loadings are dominated by data from the thigh sensor through stance and near toe-off, and that large difference features occur for the vertical vs. mediolateral accelerations near toe-off. Other areas of high loading occurred at the back primarily in the vertical direction preceding toe-off and initial contact, as well as the shank following toe-off. Again, these findings are similar to our previous work, using 3D motion capture technology, which found important features at the hip joint during early stance and toe-off and the ankle at toe-off and throughout swing [10]. Regardless, while this new IMU information provides unique insight into the biomechanical features important in these classification models, it remains difficult to convey all the high-dimensional and complex data into a simple functional or biomechanical concept. Therefore, future research should continue to investigate the complex relationships amongst many gait variables in order to provide clinicians with evidence-informed, yet simple and clinically interpretable solutions for the benefit of their patients.

In addition to its primary function of correctly identifying responders to treatment, some of the most important factors for creating a clinically viable wearable system should be time efficiency and minimal invasiveness for the patient [29]. Therefore, limiting the number of sensors, while still maintaining similar results is paramount. The current study found the classification accuracy observed with only two sensors (back and thigh: 80.0%) was not significantly different than the aforementioned three sensor array (back, thigh, and shank: 81.7%). These results suggest the three-sensor array may not be any more effective at predicting response to treatment than the simplified two-sensor array. Therefore, designing a wireless wearable system with a waist sensor and an accompanying thigh sensor may be the ideal set-up to provide clinicians with an efficient and relatively unobtrusive way to gather gait data in order to inform their treatment decisions. Not only could this system provide a “precision medicine” approach for knee OA rehabilitation, but it may also have value in other clinical groups [30].

Another potential challenge for a wearable system in a clinical setting is the training and technical skills required of the operator [29, 31]. Fortunately, given the methodology used in the current study, no complicated set-up, calibration, or computation of joint angles are required [32–34]. While more complicated methods to compute joint angles may be important in certain clinical or research applications, the current need to simply classify or sub-group gait patterns does not require this approach. Further, the current research study is supported by previous research that also demonstrates linear acceleration data from a wearable sensor can effectively classify such things as activities [35], training background [36], and other gait pathologies [37, 38]. Moreover, even with no previous experience in gait analysis, clinicians have been shown to be well equipped to accomplish this type of task [31]. Therefore, we propose that a two-sensor wearable gait analysis system could quickly and easily provide a clinician with an objective analysis to inform decision making in clinical practice.

There remain a number of potential limitations that must be acknowledged when interpreting the results of the current study. First, these remain preliminary data in the development of a wearable sensor system for improving knee OA treatment and rehabilitation. While the current results have provided important findings on creating a clinically accessible system, there remains a need to collect more data to further validate the algorithm before translating the method into clinical practice. Future research is also necessary to collect additional data to determine the true predictive capability of the algorithm in a clinical setting. Second, it should be noted that these findings relate specifically to a hip strengthening exercise intervention and should not be applied to other interventions, even those employing muscle strengthening. In other words, the current findings can help to determine if a patient is a good candidate for a hip-focused muscle strengthening program, but they do not provide any information on whether the patient would be a good candidate for a different intervention. Therefore, future research should look better identify characteristics of Non-Responders with the hope of identifying other interventions that may be more effective for this sub-group of knee OA patients. Finally, the analysis in the current study was designed to examine the overall pattern of lower limbs accelerations and high frequency accelerations that may accompany certain gait events were removed from the signal (e.g., 10 Hz low-pass cut-off filter). Therefore, high frequency components of the accelerometer signal (e.g., spectral energy, wavelet analysis) [35] that may contain useful information were not examined in the current study.

## Conclusion

This paper supports the use of wearable technology as a tool to help clinicians make evidence-informed decisions regarding optimal treatment for knee OA patients. The findings suggest that, in combination with baseline PRO measures, data from a wearable gait analysis system can successfully predict the response to a muscle strengthening intervention in a cohort of patients with knee OA. The single best placement of an accelerometer was found to be the thigh. Further, while sensors at the back, thigh, and shank provided the best classification results, a simplified two-sensor array (back and thigh) was not significantly different in performance. Although further validation is required, these findings suggest that a novel and accessible two-sensor system can be a useful precision medicine approach in helping clinicians provide the right treatment for the right patients.

## Additional file

Additional file 1: Protocol and daily training log given to knee osteoarthritis patients for the 6-week exercise intervention. (PDF 157 kb)

## Abbreviations

3D: Three-dimensional
ADL: Function in daily living
BMI: Body mass index
KOOS: Knee injury osteoarthritis outcome score
OA: Osteoarthritis
PCA: Principal component analysis
PRO: Patient reported outcome
QoL: Knee related quality of life
